# The dynamics of pain reappraisal: the joint contribution of cognitive change and mental load

**DOI:** 10.3758/s13415-020-00768-7

**Published:** 2020-01-16

**Authors:** Agnieszka K. Adamczyk, Tomasz S. Ligeza, Miroslaw Wyczesany

**Affiliations:** Institute of Psychology, Jagiellonian University, Ingardena 6, PL-30060 Krakow, Poland

**Keywords:** Pain, Cognitive reappraisal, Cognitive demand, EEG source localization

## Abstract

This study was designed to investigate the neural mechanism of cognitive modulation of pain via a reappraisal strategy with high temporal resolution. The EEG signal was recorded from 29 participants who were instructed to down-regulate, up-regulate, or maintain their pain experience. The L2 minimum norm source reconstruction method was used to localize areas in which a significant effect of the instruction was present. Down-regulating pain by reappraisal exerted a robust effect on pain processing from as early as ~100 ms that diminished the activity of limbic brain regions: the anterior cingulate cortex, right orbitofrontal cortex, left anterior temporal region, and left insula. However, compared with the no-regulation condition, the neural activity was similarly attenuated in the up- and down-regulation conditions. We suggest that this effect could be ascribed to the cognitive load that was associated with the execution of a cognitively demanding reappraisal task that could have produced a general attenuation of pain-related areas regardless of the aim of the reappraisal task (i.e., up- or down-regulation attempts). These findings indicate that reappraisal effects reflect the joint influence of both reappraisal-specific (cognitive change) and unspecific (cognitive demand) factors, thus pointing to the importance of cautiously selected control conditions that allow the modulating impact of both processes to be distinguished.

## Introduction

Pain is not a direct readout of nociceptive input (Melzack, 1999). A large body of research has demonstrated that various psychological interventions may alter the perception of its intensity and the experience of concomitant emotions. Therefore, in recent years there has been growing interest in identifying the neural pathways associated with cognitive modulation of pain experience (Edwards, Campbell, Jamison, & Wiech, 2009; Legrain et al., 2012; Tracey, 2010; Villemure & Bushnell, 2002; Wiech, 2016; Wiech, Ploner, & Tracey, 2008). While attentional modulation of pain has been extensively studied, the neuronal mechanisms that underlie the impact on pain of higher-level cognitive processes have only recently received empirical attention. Thus, in the present study we examined the unknown temporal aspect of pain modulation with the use of a cognitive reappraisal strategy by determining the modulation sequence of neural sources and tracking the observed effects in time. To our knowledge, this is the first study that has adopted this approach to study neural pain reappraisal effects.

The cognitive reappraisal strategy is regarded as one of the most effective but also one of the most cognitively complex forms of emotion regulation. It involves modifying one’s appraisal of a situation to change its emotional impact (Gross, 2014). In typical reappraisal experiments, participants are presented with emotion-inducing visual material and are instructed to generate their own alternative interpretations of negatively valenced stimuli or to detach themselves from what they see. Regulatory effects are then contrasted with a passive viewing condition in which participants are usually instructed to simply pay attention to emotional pictures. Alteration in stimuli meaning (cognitive change) is considered the primary source of the regulatory effects of reappraisal.

Within the field of pain research, however, cognitive reappraisal has been operationalized and measured differently by different researchers. One line of research investigated reappraisal processes indirectly, regarding it as a mechanism that underlies the beneficial effects of perceived control over pain (Arntz & Claassens, 2004; Mohr, Leyendecker, Petersen, & Helmchen, 2012; Salomons, Johnstone, Backonja, Shackman, & Davidson, 2007; Salomons, Nusslock, Detloff, Johnstone, & Davidson, 2014; Wiech et al., 2006). It was assumed that beliefs about the controllability of pain would change the way it is appraised, making it less threatening and subjectively more manageable even when control is not exerted or is only illusory. These studies revealed that controllable versus uncontrollable pain is indexed by increased activity in the dorsolateral PFC, ventrolateral PFC and/or ventromedial PFC (Mohr et al., 2012; Salomons et al., 2007, 2014; Wiech et al., 2006). Furthermore, perceived control over pain was associated with decreased activity in pain- and emotion-related brain regions, such as the amygdala, anterior cingulate cortex, insula, and secondary somatosensory cortex (Salomons et al., 2014; Salomons et al., 2007; Wiech et al., 2006). These findings were consistent with the vast majority of fMRI studies in which participants were instructed to reappraise emotionally arousing visual material (Kim & Hamann, 2007; Ochsner et al., 2004; Silvers, Weber, Wager, & Ochsner, 2015; Wager, Davidson, Hughes, Lindquist, & Ochsner, 2008). Therefore, it was assumed that cognitive change or change in stimulus appraisal was responsible for the changes in neural activity.

Nonetheless, inferences drawn from the aforementioned studies on reappraisal processes are indirect as none of these studies manipulated the actual meaning of pain. Thus, another line of research introduced an alternative approach: promoting participants’ active involvement in achieving a self-regulatory goal. To do so, some studies used positive self-statements, e.g., “I can stand this” (Jokic-Begic, Ivanec, & Markanovic, 2009); some involved reinterpretation of sensory experiences, e.g., imagining thermal stimulation as a hot bath (Fardo, Allen, Jegindø, Angrilli, & Roepstorff, 2015; Hampton, Hadjistavropoulos, Gagnon, Williams, & Clark, 2015; Lapate et al., 2012; Woo, Roy, Buhle, & Wager, 2015); some used verbal suggestions, e.g., convincing subjects that a procedure would improve their health (Benedetti, Thoen, Blanchard, Vighetti, & Arduino, 2013; Hovasapian & Levine, 2016); others combined some of the aforementioned methods (Denson, Creswell, Terides, & Blundell, 2014; Kalisch et al., 2005).

Down-regulating painful sensory experiences via reappraisal mitigated overestimation of remembered pain in anxious individuals (Hovasapian & Levine, 2016), reduced facial expression of pain (Hampton et al., 2015), and increased pain tolerance (Jokic-Begic et al., 2009). It also was associated with attenuation of self-reported indices of pain (Fardo et al., 2015; Hampton et al., 2015; Lapate et al., 2012), although inconsistently (Jokic-Begic et al., 2009), as well as modulation of heart rate (Kalisch et al., 2005; Lapate et al., 2012), corrugator electromyography responses (Lapate et al., 2012), and neuroendocrine activity (Benedetti et al., 2013).

At the neural level, down-regulation of painful sensory experiences resulted in modulation of activity in the medial prefrontal/anterior cingulate cortex that was accompanied by the subjective reduction of anticipatory anxiety (Kalisch et al., 2005). In studies that included two regulatory conditions and instructed participants to reinterpret their sensory experiences, down- and up-regulation was associated with decreased and increased activity in the bilateral amygdala (Lapate et al., 2012), or increased and decreased activity in the nucleus accumbens (NAcc), respectively (Woo et al., 2015). Furthermore, successful regulation, i.e., pain facilitation in up-regulation and pain inhibition in down-regulation, was mediated by functional connectivity between the nucleus accumbens and the ventromedial prefrontal cortex (Woo et al., 2015). Finally, Fardo et al. (2015) examined the effects of narrative-based mental imagery by means of EEG recording and observed modulation of the N2 potential, whose amplitude increased and decreased in the down- and up-regulation condition, respectively. In the down-regulation condition, the source of these modulations (identified for the N2 time-windows) was in the right frontal and temporal regions, whereas in the up-regulation condition it was in the left insular, middle frontal, and primary motor cortex.

In summary, both lines of study revealed that various forms of cognitive reappraisal modulate the activity of both cortical (such as the anterior cingulate cortex, primary and secondary somatosensory cortices, and insula) and subcortical brain areas (such as the amygdala and nucleus accumbens) and that this modulation originates mostly from prefrontal brain regions (such as the dorsolateral prefrontal cortex, anterior cingulate cortex, and/or ventrolateral prefrontal cortex) (Fardo et al., 2015; Kalisch et al., 2005; Lapate et al., 2012; Mohr et al., 2012; Salomons et al., 2007, 2014; Wiech et al., 2006; Woo et al., 2015). These findings suggest that, similar to other cognitive pain modulation procedures, reappraisal is associated with changes in the frontal-limbic brainstem network (Knudsen et al., 2011). Moreover, the neural activity pattern that emerges from these studies is compliant with the one observed in other paradigms that studied the impact of various forms of suggestion or induced beliefs on perceived pain, such as anticipation/placebo (Zubieta & Stohler, 2009) or hypnotic suggestion (Del Casale et al., 2015). This indicates that changing the meaning of potentially threatening or unpleasant stimuli, which is a driving force of reappraisal, may be a more universal mechanism that underlies all forms of higher-level cognitive pain modulation (Barber, 1957; Chen, 2009; van der Meulen, Kamping, & Anton, 2017; Wiech et al., 2008). However, to the best of our knowledge, none of the previous studies analyzed the exact time sequence of the observed neural modulations, which would require a much more accurate temporal resolution than the one offered by the functional resonance imaging (fMRI) that was predominantly used in these studies. Investigating this issue is of great importance as the identified brain structures participate in various mental processes, and it often is hard to distinguish their role based solely on their localization or interconnections with other brain areas. In this context, other neuroimaging methods, such as electroencephalography (EEG), that are characterized by superior temporal resolution could be useful. They could distinguish between early- and late-latency pain-modulation effects, which would help in disentangling their role in cognitive pain modulation.

Thus, the goal of our study was to investigate the temporal dynamics of higher-level cognitive modulation of pain sensations via reappraisal. Specifically, we aimed to track the activation sequence of the brain regions that are typically modulated in various belief-induction procedures (Knudsen et al., 2011; Tracey, 2010; Wiech et al., 2008). We hypothesized that we would observe modulation at both early (<300 ms) and late (>300 ms) pain-processing stages. More specifically, we assumed that the effects of reappraisal would be apparent in the modulation of the anterior cingulate cortex (ACC), the orbitofrontal cortex (OFC), and the secondary somatosensory cortex (SII)/insular cortex (IC), all of which are commonly modulated structures in cognitive pain modulation studies and have been implicated in both cognitive and affective-motivational aspects of pain (Knudsen et al., 2011; Petrovic & Ingvar, 2002; Wiech et al., 2008). Moreover, we expected that these modulatory effects would be exerted by the ventral and/or dorsolateral prefrontal cortex (PFC), both of which are regions that have previously been identified as a source of regulatory influences in pain (Mohr et al., 2012; Salomons et al., 2014) and emotion (Buhle et al., 2014) reappraisal studies. However, we did not make any specific predictions regarding the timing of the expected effects due to the repeated, bidirectional information exchange between these structures at different stages of noxious stimulus processing (Tracey & Mantyh, 2007). To obtain the latencies and location of reappraisal effects, we applied the EEG source localization method. Additionally, we collected self-reports of pain intensity, pain unpleasantness, and subjectively perceived efficiency in modulating pain experience according to the given instruction.

Although the majority of previous studies focused solely on down-regulating pain sensations using various reappraisal procedures, we included both up- and down-regulation conditions. These conditions differed from each other only in the direction of modulation attempts and were contrasted against a no-regulation condition that was characterized by significantly less intense cognitive activity. By incorporating these two regulatory conditions, we aimed to control whether reappraisal would produce specific, directional, narrative-content-based effects, or whether it would merely interfere with pain processing by redirecting attention away from the source of painful stimulation towards internal imagination-driven narration and/or by loading the executive system with a cognitively demanding reappraisal task, regardless of its content.

## Method

### Subjects

Twenty-nine healthy women volunteers participated in the study (mean age = 21.9 years; standard deviation [SD] = 1.3; range = 20–25 years). All participants were right-handed with normal or corrected-to-normal vision. No participant reported a history of pain disorders, neurological or psychiatric disorders, or substance abuse. All of them provided written, informed consent before the experiment and were informed they could withdraw from the study at any time. They received a reimbursement of €10. The study was approved by the Ethical Committee at the Institute of Psychology of Jagiellonian University and was conducted in accordance with the Declaration of Helsinki.

### Apparatus and materials

The pain stimuli were electric shocks delivered to the inner side of the left forearm through two durable stainless-steel disk electrodes 8 mm in diameter with 30-mm spacing. Each stimulus lasted 5 ms. Pain stimuli were delivered by the Constant Current High Voltage Stimulator (model DS7AH, Digitimer, Welwyn Garden City, England). Two levels of intensities were administered to the participants, corresponding to the ratings of 5 (mean = 19.17 mA, SD = 10.32 mA; range = 6–37 mA) and 7 (mean = 27.28 mA, SD = 15.11 mA; range = 8–55 mA) on a numerical rating scale (NRS). The levels were determined individually for each subject during a calibration task that was administered using PsychoPy software (Peirce, 2008) on a computer with a 61-cm LED monitor in order to control the presentation and timing of the stimuli. For EEG recording, a Biosemi Active Two EEG device was used that was equipped with 64 sensors placed on a 10-10 head cap and 4 additional electrodes placed over the eye muscles. Data processing was performed by means of EMEGS software (Peyk, De Cesarei, & Junghöfer, 2011).

### Procedure

#### Task description

Participants were asked to cognitively increase (up-regulate) or decrease (down-regulate) the upcoming sensory stimulation and to evaluate their pain experience (pain intensity, pain unpleasantness, and efficiency of pain control). At the beginning of each block, participants were delivered one of three short instructions which indicated the upcoming condition. To balance the effort of generating their own reinterpretations between the two regulation conditions, we provided participants with short instructions that encouraged them to visualize the upcoming pain in either a more negative (up-regulation condition) or more positive (down-regulation condition) way. These served as examples that could be used by participants to find their own interpretations. In the up-regulation condition, participants were instructed to imagine that pain sensations were dangerous electric shocks caused by an uninsulated cable. In the down-regulation condition, participants were instructed to imagine that pain sensations were the result of benevolent therapeutic currents (electrotherapy) whose influence would improve their overall functioning. In the case of these two regulatory conditions, participants were informed that they should build on the provided instructions (e.g., by enriching them with more details but without changing the core idea) to be able to control successfully their pain sensations. In the control condition, the instruction required the pain to be experienced naturally without any attempt to change the ongoing sensations or concomitant emotions. At the end of each block, participants were asked to rate the intensity and unpleasantness of the experienced pain and to judge their own efficiency in influencing pain sensations according to the given instruction.

Although the instructions that we used were adapted from classic reappraisal studies on emotion regulation and were modelled after so-called situation-based reappraisal (for more details on different forms of reappraisal, see Ochsner et al., 2004), changing the meaning of painful somatosensory stimulation is somewhat different than changing the meaning of unpleasant visual stimulation, which is much less aversive and much easier to reinterpret. Out of necessity, our procedure relied more on the mental imagery component, which is better suited to controlling affective experience induced in a somatosensory modality (Fernandez & Turk, 1989), than on the narrative component, which demands stimulation that is richer in detail. Furthermore, during the training we gave our participants extended reappraisal instructions to provide guidance on how to accomplish the reappraisal task correctly. However, these instructions were shorter during the main experiment and participants were encouraged to either develop their own ideas on how to reinterpret the ongoing pain or to build upon the provided examples. Thus, our procedure was somewhat different from similar belief-induction procedures, such as hypnotic or placebo suggestion paradigms (Kiernan, Dane, Phillips, & Price, 1995; Ploghaus, 1999; Wager et al., 2004), as it demanded a more active attitude towards pain.

#### Procedure

Upon arrival at the laboratory, a brief description of the experiment was provided to participants (registering brain activity while undergoing electric stimulation and performing regulatory tasks). Participants were then asked to fill in a written, informed consent form. Subsequently, the bipolar electrode was attached to the volar side of the left forearm over the median nerve; a calibration task identifying two levels of pain stimuli for each participant then started. Participants were informed that the purpose of this part of the experiment was to determine the stimulation intensity levels that would be used during the main procedure. The experimenter administered a series of trials of ascending and descending intensity (starting intensity 1 mA; step 1 mA) and recorded the participants’ responses. Participants rated the intensity of each stimulus on an 11-point numerical rating scale (NRS) ranging from 0 (no pain) to 10 (the worst imaginable pain). They then rested for approximately 2 minutes while the experimenter calculated the average values (mA levels) corresponding to 5 and 7 (the values that were chosen for the experimental procedure) on the NRS. Then, written and oral instructions describing the experimental task were provided, and a brief training session consisting of six blocks (2 for each condition) started. Descriptions used in the training session were as follows. For the up-regulation condition (UPREG): “INCREASE PAIN. Imagine that the ongoing pain is a result of a strong electric shock caused by a noninsulated cable. Think how dangerous this shock is for your life and the great pain it causes.” For the down-regulation condition (DWREG): “DECREASE PAIN. Imagine that the ongoing stimulation is a result of therapeutic currents, so-called “electrotherapy,” whose influence will improve the harmonized functioning of your organism, streamline blood circulation, oxygenate the tissues, and allow you to relax better.” For the control condition (NOREG): “MAINTAIN PAIN. Experience pain naturally, without attempting to change the ongoing sensations or emotions.” All participants reported that the six training blocks were sufficient for the task to be understood. After the training session the EEG equipment was mounted.

**Fig. 1:**
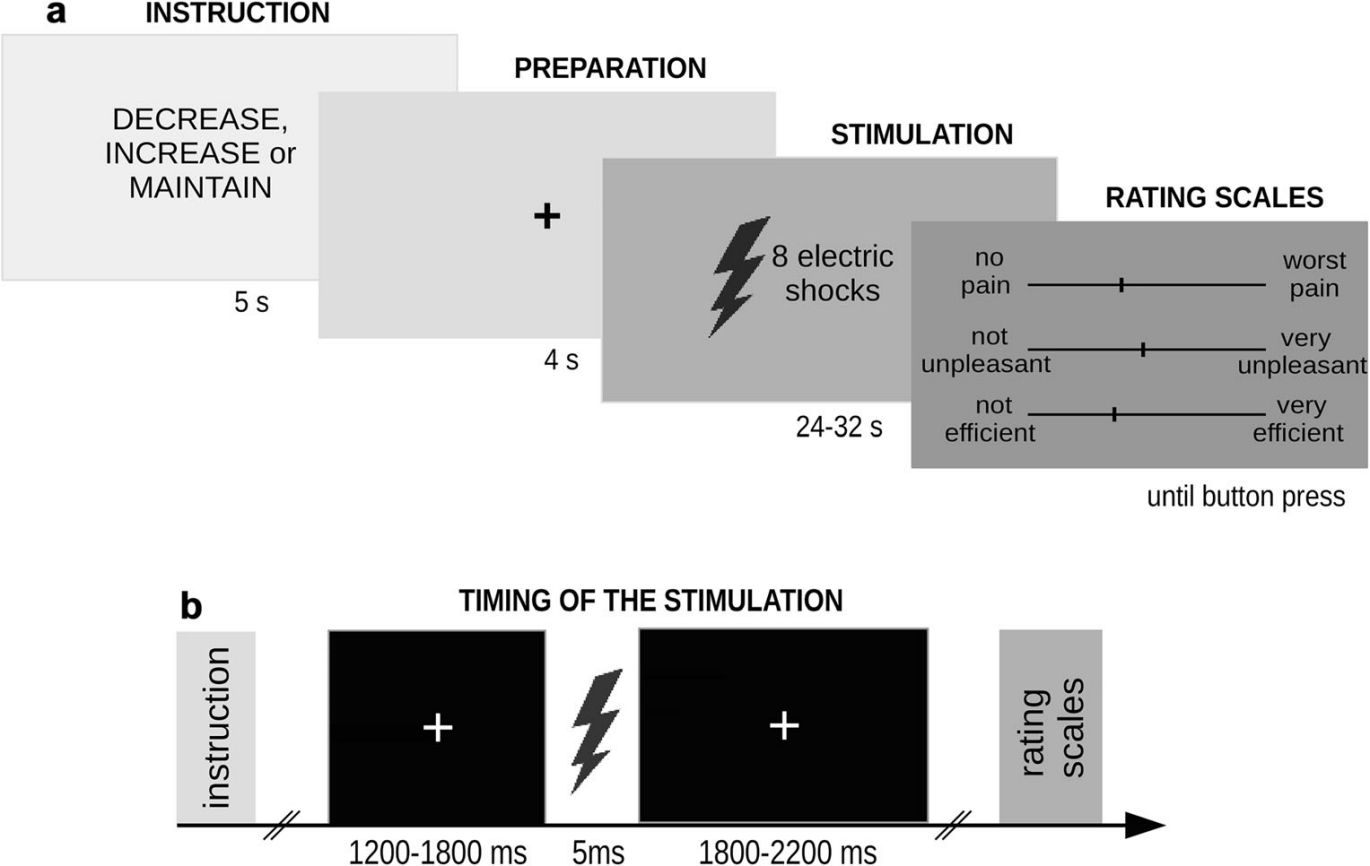
**a** Timeline of a single block; **b** Timeline of the stimulation.

The experimental task consisted of 30 blocks, 10 for each condition (Fig. 1A). Each block started with a shortened version of one of the three instructions used in the training session, informing the participant of the upcoming condition (i.e., the up-regulation, down-regulation, or no-regulation condition). Instructions were as follows. For the up-regulation condition: “INCREASE PAIN. Imagine that pain sensations are dangerous electric shocks being caused by a noninsulated cable.” For the down-regulation condition: “DECREASE PAIN. Imagine that pain sensations are the result of benevolent therapeutic currents (electrotherapy), whose influence will improve your overall functioning.” For the no-regulation condition: “MAINTAIN PAIN. Experience pain naturally, without attempting to change the ongoing sensations or concomitant emotions.” The instruction was presented for 5 s and was followed by a short preparation period (4 s). Then, a set of 8 stimuli of one predefined intensity (corresponding to the subject’s rating of 5 or 7 on the NRS) was delivered, separated from each other by a 3–4 s interstimulus interval (Fig. 1B). The duration of each stimuli was 5 ms. Importantly, participants were not informed that only two levels of pain intensity were administered. Blocks were concluded with rating scales presented on the horizontal visual-analogue scales (VASs). On the first scale, participants rated the experienced painfulness (“How much pain did you feel?”; 0 = no pain, 100 = the worst imaginable pain). On the second scale, they rated the experienced unpleasantness (“How unpleasant did you feel?”; 0 = no unpleasantness; 100 = the worst unpleasant pain). Finally, in the up- and down-regulation blocks, participants were asked to rate their efficiency in influencing the pain experience (“How efficient were you in influencing your sensations?”; 0 = not efficient; 10 = very efficient). A 1-s interval separated the consecutive blocks. The blocks were presented with one of two pseudo-randomized orders to counterbalance order effects: N N D, D, U, U, N N U, U, D, D, U, U, D, D, N N U, U, D, D, N N D, D, U, U, N N or D, D, N N U, U, N N U, U, D, D, D, D, U, U, N N D, D, N N U, U, N N U, U, D, D (where N corresponds to the no-regulation condition, D to the down-regulation condition, U to the up-regulation condition).

### Data analysis

#### EEG preprocessing and data analysis

The sampling was set to 256 Hz. The signal was filtered using 0.1-Hz high-pass and 46-Hz low-pass zero-phase filters and referenced to the averaged potential from all headcap electrodes. Ocular artifacts were corrected using Biosig toolbox (Schlögl, Vidaurre, & Sander, 2011): the signal was epoched in −100 to 1,000 ms time windows relative to electric stimulation onset; it was baseline corrected using the mean prestimulus value in the range of −100 to 0 ms. Artifact rejection using the method for statistical control of artifacts in high-density EEG/MEG data then followed (SCADA; Junghöfer, Elbert, Tucker, & Rockstroh, 2000); it included detection of individual channel and global artifacts, interpolation of rejected sensors, and verification of the stability of the trials across the whole recording by computing their variance. Remaining epochs were averaged across conditions. Finally, the L2 minimum-norm inverse modeling method (Hämäläinen & Ilmoniemi, 1994) was used to estimate the activity of cortical sources contributing to the scalp signal. The spherical shell consisting of 350 evenly distributed dipole pairs was used as a head model with a radius of 90% of the averaged head (roughly corresponding to gray matter depth), with the Tikhonov regularization parameter k set to 0.1. The resulting topography maps were projected onto a realistic brain geometry (Bröckelmann et al., 2013). To reveal spatiotemporal differences in brain activation between reappraisal conditions, a nonparametric cluster-mass statistical procedure with correction for multiple comparisons was applied (Maris & Oostenveld, 2007). This approach is entirely data driven and yields the spatial and temporal extent of the significant effect without prior assumption of particular ROIs or time windows. According to this procedure, all t-values which exceeded critical alpha-levels (*p* = 0.05; sensor-level criterion) were summed across neighboring dipoles (located within an angle of 120 degrees from the vertex, which roughly reflects cortical dipoles) and adjacent time points in order to form spatiotemporal clusters comprised of electrodes/samples, which reached the assumed significance level. The number of these electrodes/samples together with their effect strength are considered as cluster mass, which together describe the spatiotemporal extent of the cluster. For the sake of clarity, we will refer to the masses as neural activations. Then, the masses of all obtained clusters were compared against a random permutation cluster-based alpha-level (cluster-level criterion; *p* = 0.05) that was established via Monte Carlo simulations (1,000 permutations) (Wessing, Rehbein, Postert, Fürniss, & Junghöfer, 2013). The only clusters reported were those whose spatiotemporal extent reflected by the obtained masses exceeded the critical cluster-level threshold. Finally, the resulting clusters were subjected to ANOVA statistics to determine the effects of reappraisal condition and intensity. Additionally, a classic ERP analysis was performed by which we estimated the effect of condition on the N2-P2 mean amplitude difference at the typically analyzed vertex Cz electrode (Albu & Meagher, 2019; Bromm & Lorenz, 1998; Meier, Klucken, Soyka, & Bromm, 1993; von Mohr, Krahé, Beck, & Fotopoulou, 2018) using 100–130 ms and 220–260 ms time-windows, respectively. To examine the impact of different tasks on the N2-P2 amplitudes, a one-way repeated measures ANOVA was conducted with the Instruction (down-regulation, no-regulation, up-regulation) as the repeated-measure factor.

#### Subjective ratings analysis

In order to examine the impact of the different tasks (up-regulation, down-regulation, no-regulation condition) on subjective ratings of perceived pain intensity and unpleasantness and with respect to two stimulus intensities, two-way repeated measures ANOVAs were conducted. To assess mean differences in subjective efficiency ratings between the two regulatory conditions (up- and down-regulation) and with respect to the two stimulus intensities (moderate and high), a two-way repeated measures ANOVA was applied. Statistical significance was set at *p* < 0.05 and effect sizes were calculated using the partial η^2^. The Greenhouse-Geisser correction was applied if needed, and adjusted *p*-values are reported. In the case of significant effects, the Bonferroni correction was used for post-hoc comparisons.

## Results

### Subjective ratings data

**Fig. 2:**
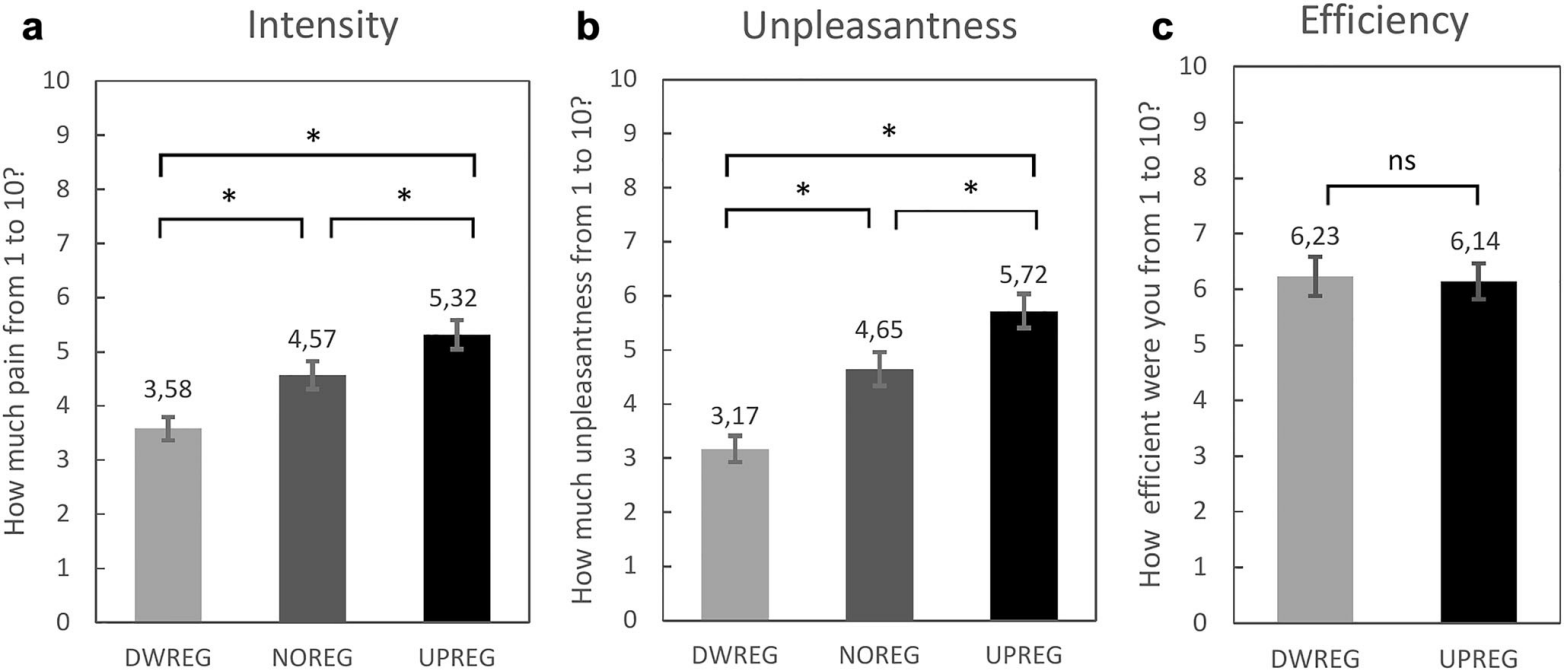
Subjective ratings for down-regulation condition (DWREG), no-regulation condition (NOREG) and up-regulation condition (UPREG). A Mean and SEM of pain intensity ratings; B Mean and SEM of pain unpleasantness ratings; C Mean and SEM for efficiency ratings.

Participants reported significant differences in perceived pain intensity and unpleasantness according to the given instruction. Compared with no-regulation (NOREG), the up- (UPREG) and down-regulation (DWREG) conditions were associated with significantly increased or decreased intensity and unpleasantness ratings, respectively (Figs. 2A and 2B). The main effect of instruction for subjectively perceived pain intensity ratings corresponded to F(2,56) = 66.10, *p* < 0.001, partial η^2^ = 0.70. The instruction main effect for subjective unpleasantness ratings was F(1.64,45.9) = 61.85, *p* < 0.001, partial η^2^ = 0.69 (Figure 2).

Furthermore, stimulus intensity influenced both perceived pain intensity and unpleasantness ratings, irrespective of the given task. When the applied stimulus was of moderate intensity, the mean subjective intensity rating was 3.69 (SE = 0.23) and the mean subjective unpleasantness rating was 3.74 (SE = 0.26). Conversely, when the applied stimulus was of high intensity, the mean subjective intensity rating was 5.29 (SE = 0.26) and the mean subjective unpleasantness rating was 5.51 (SE = 0.38). The main effect of stimulus intensity for subjective intensity ratings was F(1,28) = 97.42, *p* < 0.001, partial η^2^ = 0.78. The main effect of instruction for subjective unpleasantness ratings was F(1,28) = 36.53, *p* < 0.001, partial η^2^ = 0.57. However, the interaction effects between instruction and stimulus intensity for both perceived pain intensity and unpleasantness ratings yielded insignificant results: F(2,56) = 2.30, *p* = 0.11, and F(1.15,32.31) = 0.20, *p* = 0.691, respectively.

Participants also were instructed to rate their efficiency in influencing pain sensations according to the given instruction. The results revealed that the perceived efficiency ratings for down- and up-regulation did not differ: F(1,28) = 0.449, *p* = 0.508). Participants reported feeling equally capable of down-regulating (mean rating = 6.23, SE = 0.35) and up-regulating (mean rating = 6.15, SE = 0.32) their sensory experience (Fig. 2C). However, further analyses revealed that participants’ efficiency ratings were influenced by stimulus intensity as there was a significant interaction between instruction and stimulus intensity: F(1,28) = 37.53, *p* < 0.001, partial η^2^ = 0.57. All comparisons yielded significant results: participants reported being more efficient (*p* < 0.001) at down-regulating moderate-intensity stimuli (mean rating = 6.50, SE = 0.34) than at up-regulating them (mean rating = 5.79, SE = 0.32) or at down-regulating high-intensity stimuli (*p* = 0.003); they were more efficient (*p* = 0.015) at up-regulating high-intensity stimuli (mean rating = 6.43, SE = 0.34) than at down-regulating them (mean rating = 6.04, SE = 0.37) or at up-regulating moderate-intensity stimuli (*p* < 0.001).

### EEG data

**Fig. 3:**
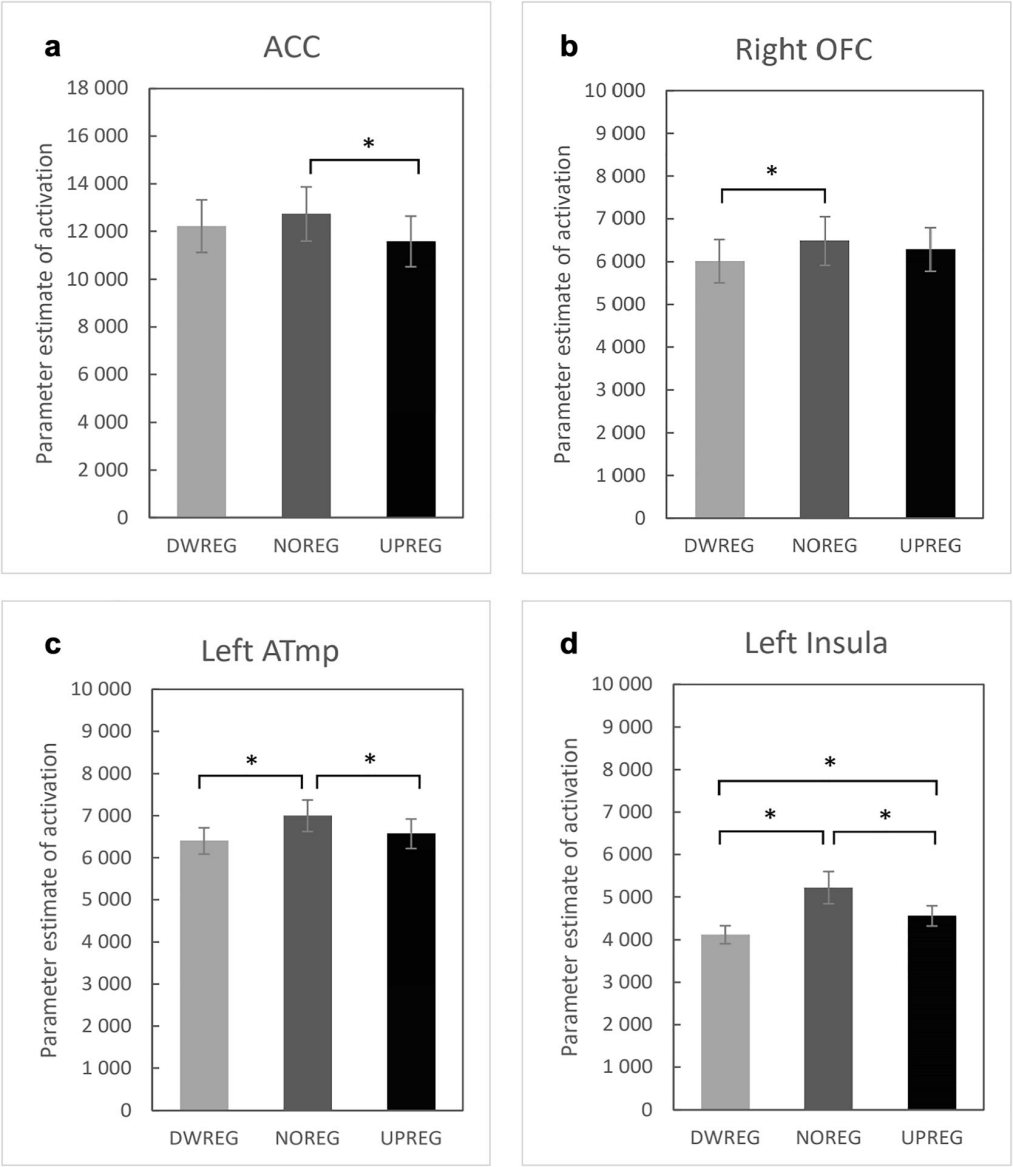
Main effects of instruction for clusters of neural activation in down-regulation condition (DWREG), no-regulation condition (NOREG), and up-regulation condition (UPREG).

**Fig. 4:**
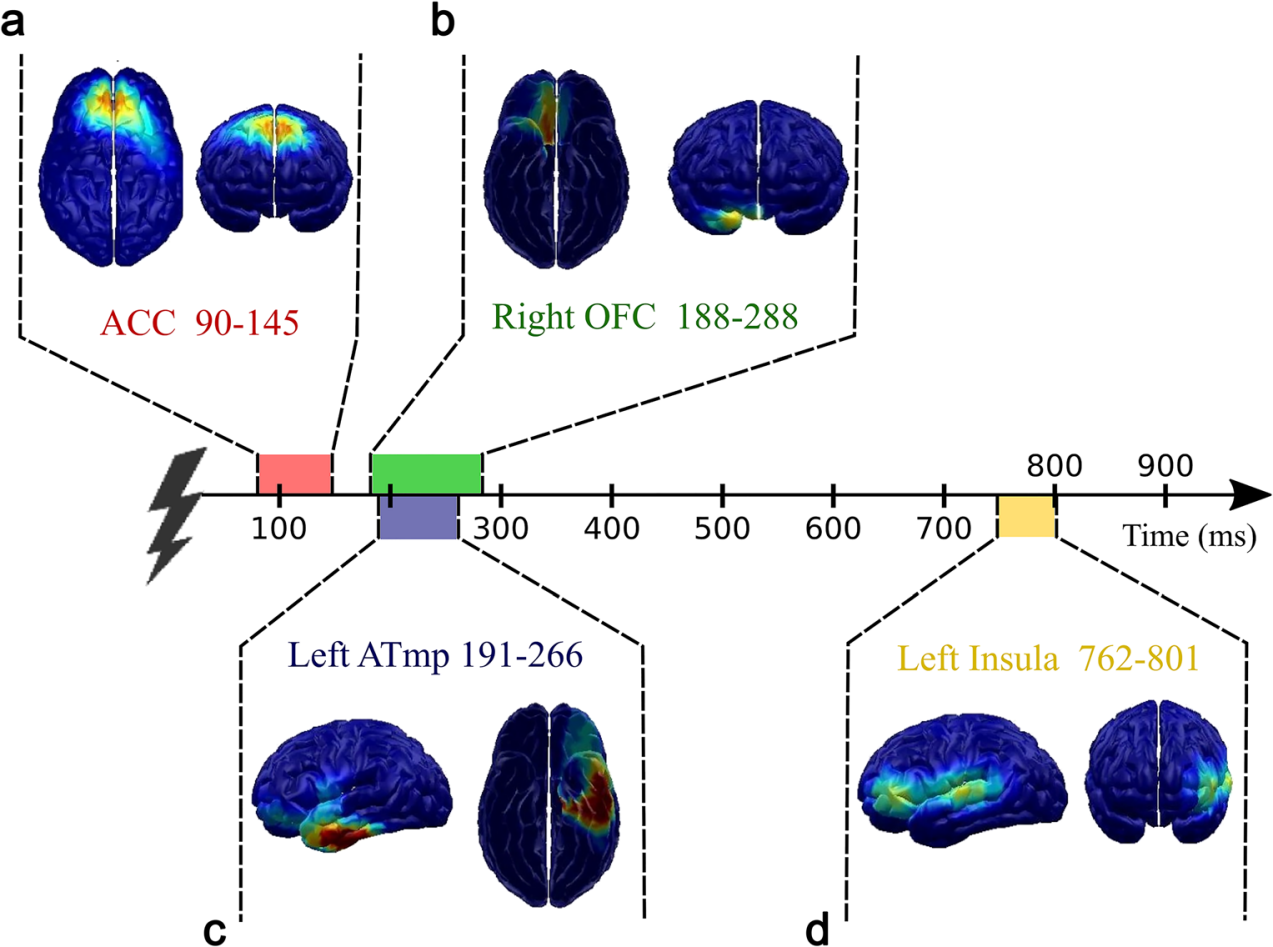
EEG clusters showing a significant main effect of instruction with respect to their timing.

**Fig. 5:**
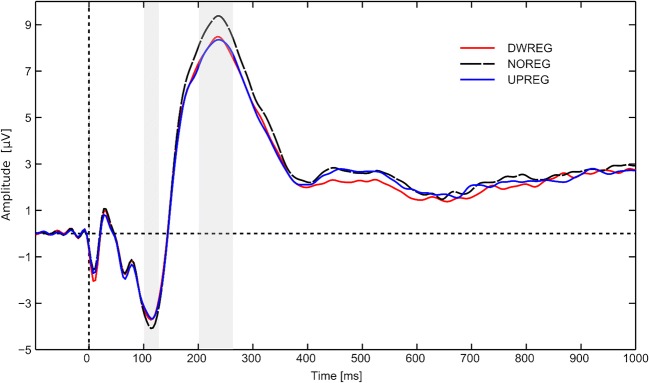
Grand averaged waveforms at the vertex (Cz electrode) showing the N2-P2 complex evoked by down-regulation condition (DWREG), no-regulation condition (NOREG), and up-regulation condition (UPREG).

Four brain clusters were found in which the main effect of the reappraisal condition was observed: the anterior cingulate cortex (ACC) in the early 90–145 ms window relative to stimulus onset (*F*(2,56) = 8.45; *p* = 0.001; Figs. 3A and 4A); the right orbitofrontal cortex (ROFC) in the 188–288 ms time window (*F*(2,56)=4.61; *p* = 0.017; Figs. 3B and 4B); the left anterior temporal area (LATmp) in the 191–266 ms window (*F*(2,56)=16.49; *p* < 0.001; Figs. 3C and 4C); and the left frontotemporal area, identified as the left insula (LIns) in the late 762–801 ms time window (*F*(2,56) = 14.62; *p* < 0.001; Table 1 Figs. 3D and 4D). Post-hoc comparisons revealed that all the identified clusters showed a similar pattern of activation changes, with the highest value observed in the control condition (NOREG) compared to at least one of the regulation (UPREG or DWREG) instructions. For the ACC, higher activation was recorded in the NOREG compared with the UPREG condition (*p* = 0.003). For the ROFC, activation was higher in the NOREG than in the UPREG condition (*p* = 0.011). For the LATmp, activation was higher in the control condition compared to both the UPREG and DWREG conditions (*p* = 0.001). Interestingly, post hoc tests yielded all significant comparisons for the LIns. Higher activation was observed for the control condition compared with the up-regulation condition (*p* = 0.025) and the down-regulation condition (*p* < 0.001). Additionally, there was a significant difference between the regulation conditions, with greater activity in the UPREG condition (*p* = 0.003). The effect of intensity was also observed for all clusters, with higher activation for more intensive stimulation: *F*(1,28) = 76.67; *p* < 0.001 for the ACC cluster; *F*(1,28) = 22.73; *p* < 0.001 for the ROFC area; *F*(1,28) = 57.06; *p* < 0.001 for the LATmp; *F*(1,28) = 6.79; *p* = 0.015 for the left insular cluster. Finally, our analysis of the ERP components revealed that reappraisal tasks successfully modulated the N2-P2 mean amplitude difference: *F*(2,56) = 13.25, *p* < 0.001; η^2^= 0.314 (Fig. 5). This is consistent with the neural modulation pattern of the identified clusters. Post hoc comparisons confirmed that both down- (M = −11.58; SD = 5.88) and up-regulation (M = −11.59; SD = 6.00) tasks reduced the N2-P2 mean amplitude difference (*p* < 0.001) compared with the no-regulation task (M = −12.82; SD = 5.99), but there was no significant difference between them (*p* = not significant [ns]).

**Table 1.** Neural activations (masses) of clusters (M±SE) by condition

	DWREG	NOREG	UPREG
*ACC (90–145 ms)*	122.22 ± 11.10	127.30 ± 11.46	115.83 ± 10.68
*Right OFC (188–288 ms)*	60.10 ± 5.12	64.81 ± 5.76	62.83 ± 5.16
*Left ATmp (191–266 ms)*	63.99 ± 3.14	69.98 ± 3.81	65.70 ± 3.56
*Left Ins (762–801 ms)*	41.17 ± 2.42	52.20 ± 4.10	45.57 ± 2.55

*ACC* Anterior cingulate cortex, *OFC* Orbitofrontal cortex, *ATmp* Anterior temporal area, *NOREG* No regulation condition, *UPREG* Upregulation condition, *DWREG* Downregulation condition. The values in the table are divided by a factor of 100.

## Discussion

Using a cutting-edge EEG source localization method, the goal of the present study was to examine the temporal dynamics of reappraisal effects on neural processing of nociceptive input and its influence on the subjective perception of pain. Participants were asked to reinterpret the meaning and source of the upcoming painful stimulation using a narrative-based mental imagery task (down- and up-regulation condition) or to “allow their emotions and thoughts to arise freely” (no-regulation condition) following the application of pain stimuli. By incorporating and contrasting the two regulatory conditions, we aimed to discern whether reappraisal would produce specific narrative-content-based effects that increased and decreased pain experience according to the given instruction, or whether it would merely interfere with pain processing by redirecting attention away from the source of painful stimulation towards internal imagery-driven narration and/or by increasing the mental load induced by a cognitively demanding reappraisal task, regardless of its content.

We found robust evidence that reappraisal modulated neural processing of nociceptive input from as early as 100 ms onwards: the main effect of instruction was identified for four clusters of neuronal activation localized within the anterior cingulate cortex (90–145 ms), the right orbitofrontal cortex (188–288 ms), and the left anterior temporal region (191–266 ms), all of which can be considered early latency effects, and the left insula (762-801 ms), which is a relatively late latency effect. Neural modulation was accompanied by modulation of subjective ratings: participants reported reduced pain and unpleasantness in the down-regulation condition and, conversely, enhanced pain and unpleasantness in the up-regulation condition relative to the no-regulation condition. Furthermore, they assessed themselves as being equally successful in decreasing and increasing pain.

### Temporal sequence of neural modulation

#### Early latency effects (<300 ms)

##### Anterior cingulate cortex

The earliest modulatory effects of reappraisal were reflected in ACC activation (90–145 ms; Fig. 4A). The magnitude of its response was significantly smaller in the up-regulation condition compared with the no-regulation condition, but the difference between the up- and down-regulation conditions was non-significant. Although the ACC is one of the most consistently activated structures in both emotion (Phan, Wager, Taylor, & Liberzon, 2002) and pain studies (Peyron, Laurent, & García-Larrea, 2000), its function differs significantly depending on the ACC subdivision and the timing of observed effects. The dorsal-caudal ACC subdivision serves a function that is common to pain, negative affect and cognitive control, detecting events or internal states and signaling a need to intensify or redirect attention or to strengthen top-down control in order to prevent a potential threat or future error (Botvinick, Carter, Braver, Barch, & Cohen, 2001; Shackman et al., 2011). The ventral-rostral subdivision of the ACC, which exchanges reciprocal connections with the amygdala, has been implicated in the perception of pain unpleasantness in both experimental (Rainville, Duncan, Price, Carrier, & Bushnell, 1997) and clinical studies (Foltz & White, 1962). Importantly, attenuation of ACC activity has been observed in studies examining the effects of perceived control over pain (Mohr et al., 2012; Salomons et al., 2007, 2014; Wiech et al., 2006) and reappraisal of pain sensations via mental detachment (Kalisch et al., 2005), thus indicating that cognitive manipulation of pain meaning was capable of decreasing its motivational value. On the other hand, decrease of pain-evoked activity in the ACC has been reported in studies examining the regulatory effects of attention-demanding cognitive tasks that were unrelated to the meaning of nociceptive stimulation (Bantick et al., 2002; Frankenstein, Richter, McIntyre, & Rémy, 2001; Rémy, Frankenstein, Mincic, Tomanek, & Stroman, 2003; Seminowicz & Davis, 2007; Valet et al., 2004; Wiech et al., 2005), thus suggesting that tasks that are sufficiently engaging are capable of diminishing pain-related salience. Finally, activity of the ACC also is sensitive to expectancy manipulations; whereas expectations of high stimulus intensity cause an increase in the anticipatory activity of the ACC, expectations of low stimulus intensity decrease its activity (Fairhurst, Wiech, Dunckley, & Tracey, 2007; Koyama, McHaffie, Laurienti, & Coghill, 2005). The variety of procedures that involve ACC modulation suggests that it is engaged in different pain processes; however, these are hard to distinguish given the poor temporal resolution of fMRI. Although the limited spatial resolution of EEG does not unequivocally show which of the ACC subdivisions contributed to the observed effect, on the basis of the timing of the neural modulation effect we can hypothesize which psychological process was modulated. On the one hand, considering the successive modulation pattern of the adjacent limbic structures, it is possible that it reflected the reduced emotional responding that took place in its “affective” subdivision. On the other hand, considering that modulation was observed very early (90–145 ms), it most probably originated in the ACC’s “cognitive” subdivision and reflected involuntary redirection of attention away from the cognitive task and towards the salient and threatening nociceptive stimulation (Dowman, 2004; Eccleston & Crombez, 1999; Peyron et al., 1999, 2000) that took place in the down- and no-regulation conditions but was absent in the up-regulation condition, whose task might have encouraged participants to monitor the onset of nociceptive stimulation. Similar timing of ACC modulation was observed in a study in which participants were subjected to a simultaneous painful conditioning and distraction procedure (Moont, Crispel, Lev, Pud, & Yarnitsky, 2012).

##### Orbitofrontal cortex

Within the 188–288ms time window, we observed the hypothesized modulation of the right orbitofrontal cortex (Fig. 4B), which showed diminished activity in the down-regulation condition compared with the no-regulation condition but did not differ from the up-regulation condition. Orbitofrontal cortex (OFC) activation has been observed during the experience of a wide range of affective states (Berridge & Kringelbach, 2008; O’Doherty, Kringelbach, Rolls, Hornak, & Andrews, 2001), including pain (Tracey, 2010; Wiech, Seymour, Kalisch, Stephan, et al., 2005). Moreover, it has been suggested that the activity of the right lateral OFC represents the magnitude of expected costs or the punishing value of the stimulus (Grabenhorst & Rolls, 2011; O’Doherty et al., 2001), which is integrated with and informed by relevant contextual information and thus produces a conceptually enriched affect (Roy, Shohamy, & Wager, 2012). Furthermore, because it is densely connected with regions that code for affective components of pain, such as the ACC, the insula, and the amygdala (Kringelbach & Rolls, 2004), activation of the lateral OFC is commonly correlated with the subjective unpleasantness of pain experience (Plassmann, O’Doherty, & Rangel, 2010; Rolls et al., 2003; Seymour et al., 2005; Wiech et al., 2005) and is thought to reflect emotional-motivational appraisal of pain (Seymour et al., 2005; Wiech et al., 2006; Wiech et al., 2005). Thus, in line with other studies utilizing aversive stimulation, the right OFC deactivation observed in the down-regulation condition most probably reflected attenuated emotional responding (Rolls, 2003; Seymour et al., 2005; Wiech et al., 2005). The observed timing, which resembles the pattern of OFC responses during valence discrimination (starting around 100 ms post-stimulus and reaching a maximum at around 180 ms (Kawasaki et al., 2001) further supports this interpretation. It is plausible that at this stage of processing the intrinsically aversive value of the pain stimulus was integrated with instruction-appropriate conceptual framing, which resulted in reevaluation of its meaning (cognitive change). Conversely, the lack of a similar increase of neural response in the up-regulation condition supports the role of nonspecific cognitive activity in the attenuation of affective responding that is observed in typical emotional control tasks used in laboratory procedures.

##### Anterior Temporal Lobe

In the 191–266 ms time window, we found modulation of the left anterior temporal area (Fig. 4C), whose activity was attenuated in both regulation conditions compared with the no-regulation condition. The limited spatial resolution of our method does not allow for unequivocal conclusions about the exact location of the source structure, which could be either the anterior part of the temporal lobe or the amygdalar complex. The latter possibility could be supported by the well-known role of this structure in representing the motivational value of stimulation (especially for negative valence) and the relatively early onset of this effect (Sabatinelli, Lang, Bradley, Costa, & Keil, 2009). The amygdala plays a critical role in emotional response to pain and in pain modulation; it integrates nociceptive information with internal (e.g., affective (Roy, Piche, Chen, Peretz, & Rainville, 2009)) and external (e.g., environmental (Atlas, Bolger, Lindquist, & Wager, 2010)) states, thus facilitating or inhibiting pain processing in different areas of the brain. Pain-related amygdala activity also is susceptible to conceptual manipulations of the meaning of pain; its activity has been shown to increase or decrease depending on the reappraisal instructions (Lapate et al., 2012) and is modulated by the manipulation of perceived control over pain (Salomons et al., 2007, 2014; Wiech et al., 2006). On the other hand, the temporal poles, which are considered by some authors to constitute an “extended limbic system,” also are related to processing of emotional stimuli (Olson, Plotzker, & Ezzyat, 2007). In line with the current results, activity of the left anterior temporal cortex has been shown to increase together with subjective ratings of negative emotions in response to negatively valenced stimuli (Wyczesany & Ligeza, 2014). Consistently with the activation of the right OFC, for which a similar modulation pattern was observed in the overlapping time window (188–288 ms), the pattern of amygdala/LATmp modulation could reflect the process of affective elaboration of pain stimuli. Such temporal synchrony with the OFC indicates a process of valence elaboration, which is consistent with the current views on the role of these regions (Pessoa & Adolphs, 2010) and the typical timing of amygdala modulation (Sabatinelli et al., 2009).

#### Late latency effects (>300 ms)

Last but not least, we observed modulation of the left insular cortex within the 762–801 ms time window (Fig. 4D). Interestingly, all comparisons yielded significant effects: activation was lowest in the down-regulation condition and highest in the no-regulation condition. The insula often is bilaterally activated during noxious stimulation (Coghill et al., 1994); however, its modulation is observed in both early and late pain processing stages, suggesting that multiple functions are served by this structure (Moont, Crispel, Lev, Pud, & Yarnitsky, 2011; Moont et al., 2012). While the anterior insula tracks affective-motivational aspects of pain processing and contributes to interoceptive awareness (Craig, 2002), stimulus intensity is coded in the posterior insula (Craig, Chen, Bandy, & Reiman, 2000). It has been proposed that the insula integrates information about one’s current bodily and affective states with higher-level cognitive information concerning the current (task) goals and afferent sensory processing (Starr et al., 2009). Moreover, the anterior insular cortex has been identified as an important node of the salience network, which includes the dorsal ACC and other subcortical limbic regions (Lindquist, Wager, Kober, Bliss-Moreau, & Barrett, 2012; Uddin, 2015). The fact that the anterior insula is implicated in both affective integration and cognitive control indicates that it promotes reappraisal success; indeed, modulation of this structure has been consistently observed across various reappraisal studies (Fardo, Allen, Jegindø, Angrilli, & Roepstorff, 2015; Salomons, Johnstone, Backonja, Shackman, & Davidson, 2007; Salomons, Nusslock, Detloff, Johnstone, & Davidson, 2014; Starr et al., 2009; Wiech et al., 2006; Woo, Roy, Buhle, & Wager, 2015). Late deactivation of the left insula (>550 ms post-stimulus) also was reported in an EEG study with pain modulation using distraction (Moont et al., 2012). Considering the relatively late insular activation (~800 ms), its integrative function, and its similar pattern of activation to those reported above, it is possible that modulation of the insular cortex observed in our study reflected the resultant activity of all the aforementioned structures. Here, the signals concerning emotional state, current task goals, and incoming sensory information converged, replicating the general pattern of modulation observed in other limbic areas that is apparent in an overall decrease in neural activity observed in (up- and down-) regulation versus no-regulation conditions, but this time accompanied by an increase in neural activity in the up- versus down-regulation condition.

### Question of the specificity of the instruction effect

In summary, we found extensive evidence that reappraisal modulated neural processing of nociceptive input at both early and late stages of pain processing. The observed pattern of neuronal activation indicates that reappraisal influenced the affective-evaluative aspect of pain processing by modulating the activity of the “rostral limbic system” (Devinsky et al., 1995).

However, contrary to our expectations and the available literature, we observed a non-specific attenuation of neural response to pain in one or both regulatory conditions compared with the no-regulation condition. Apart from the left insula, the neural response to pain in the down-regulation condition was indistinguishable from the neural response in the up-regulation condition. Assuming that the activity of the identified neural sources reflected the depth of processing of the nociceptive input at its different stages, this consistent modulation pattern appears to indicate that the attenuating effect of cognitive reappraisal can largely be considered nonspecific.

The above interpretation appears to be supported by several pieces of independent evidence. First, although all identified clusters were localized blindly, i.e., without previous assumptions or predetermination of regions of interest (ROIs), their location is compliant with structures that show a consistent increase in activity when processing nociceptive input (Apkarian, Bushnell, Treede, & Zubieta, 2005; Peyron et al., 2000; Tracey & Mantyh, 2007; Wager et al., 2013). This finding suggests that their activity can be treated as a measure of the neural response to pain, yet it obviously reflects its various separate subprocesses that are engaged in generating an appropriate, context-dependent response to pain. Furthermore, neuroanatomical studies have demonstrated that all four structures are densely (and often bidirectionally) interconnected and that by sending and receiving projections from one another they are capable of affecting each other’s activity when processing nociceptive input (Beckmann, Johansen-Berg, & Rushworth, 2009; Craig, 2009; Kringelbach & Rolls, 2004). This observation could explain the observed consistency of the modulation pattern of each structure’s activity (NOREG > DWREG, NOREG > UPREG, DWREG ≠ UPREG). Finally, it has been suggested that the rostro-dorsal ACC, lateral OFC, amygdala, and anterior insula constitute the brain’s negative appraisal system, whose activity indexes the effectiveness of negative emotion regulation via reappraisal (Wager et al., 2008). This finding lends further support to the assumption that activation of all four identified brain regions reflects the intensity of processing of nociceptive input and thus might be treated as a proxy for the regulatory effects of reappraisal.

### Mechanism that drove the reappraisal effects

Our results suggest that in addition to cognitive change, regulatory effects are also driven by other factors that are nonspecific to the cognitive reappraisal strategy per se. This finding stands in contrast to other studies that examined the neural mechanisms that underlie cognitive modulation of pain using both “passive” (i.e., examining effects of perception of pain controllability; (Mohr et al., 2012; Salomons et al., 2007, 2014; Wiech et al., 2006)) and “active” reappraisal procedures (i.e., instructing participants to actively achieve a self-regulatory goal; (Fardo et al., 2015; Kalisch et al., 2005; Lapate et al., 2012; Woo et al., 2015)). The question of why this pattern of results emerged in our study thus arises. After careful scrutiny of the current literature, we suggest that this discrepant finding could have arisen due to several factors. First of all, some studies intermixed different methods of reducing emotional responses to pain (Denson et al., 2014; Kalisch et al., 2005), which ultimately makes it impossible to assess which of them actually contributed to the observed modulatory effects. For instance, Kalisch et al. (2005) instructed their participants not only to detach themselves from the painful somatosensory stimulation by imagining that they were in their “special” and safe place but also to generate positive self-statements and to relax their muscles by deploying a technique that they were taught at the beginning of the experiment.

Secondly, although a large body of literature indicates that perceived controllability over pain results in a robust modulation of the pain experience, the conclusions that these benevolent effects are primarily driven by cognitive change are inferred indirectly as none of these studies actually manipulated the meaning of pain (Mohr et al., 2012; Salomons et al., 2007, 2014; Wiech et al., 2006). It is thus conceivable that these effects could, for instance, originate from a more generalized increase in self-control, not from the cognitive reevaluation of the threatening value of stimuli.

Third, classic reappraisal procedures (in which participants are actively engaged in the regulation of their affective states) differ from studies on the perceived controllability of pain in one other respect: they are far more cognitively demanding and effortful, because participants are usually required to invent their own ideas of how to reinterpret a given emotion-evoking stimulus and subsequently to monitor its successfulness in reducing or augmenting their affective state. As a consequence, reappraisal engages numerous mental processes, including 1) elaboration of an emotion-provoking situation; 2) finding an adequate reinterpretation of the situation’s meaning; 3) maintaining the new interpretation in working memory; 4) tracing one’s own affective state that arises in response to the modified interpretation; and 5) adjusting the reinterpretation if necessary. Although neglected, all these accompanying processes have the potential to significantly decrease emotional responding even before the conceptual reinterpretation takes place.

Hence, as a cognitive reappraisal task engages numerous mental processes (Ochsner, Silvers, & Buhle, 2012) and is one of the most cognitively demanding means of regulating one’s emotions (Sheppes, Catran, & Meiran, 2009; Sheppes & Meiran, 2008), it appears plausible that some of these processes have the potential to attenuate emotional response even before cognitive change takes place. As a matter of fact, our previous study was exclusively devoted to investigating this issue, albeit in the visual domain (Wyczesany & Ligeza, 2017). For this reason, we designed a “retro” task that was aimed to be as similar to a reappraisal task as possible (in terms of cognitive demand and the required level of cognitive elaboration) but that would lack the reappraisal-specific process of cognitive change. Our results revealed that emotional responding was reduced by both tasks, regardless of whether the cognitive change was present or was substituted with a similarly engaging task related to the picture content. This finding indicates that the cognitive effort associated with understanding and elaborating the content of emotional stimuli (i.e., the essential processes that precede successful reappraisal) can significantly contribute to the attenuation of emotional responses alongside cognitive change.

Taking all this into consideration, the current results seem to extend these findings to the pain domain. The observed modulation pattern (NOREG > DWREG, NOREG > UPREG, DWREG ≠ UPREG) may have resulted from the additive influence of both cognitive change (that reduced or escalated pain processing according to the provided instruction) and the unspecific cognitive load that was imposed on the cognitive system by performing a cognitively demanding reappraisal task that attenuated neural processing in both regulation conditions compared with the less cognitively engaging no-regulation condition. The interaction of these two processes was probably best reflected in the activation pattern of the left insula, for which comparisons between all conditions yielded significant effects, with the highest activation in the no-regulation condition and the lowest in the down-regulation condition (NOREG > UPREG > DWREG). It seems that while in the down-regulation condition both cognitive change and unspecific cognitive load operated jointly to attenuate pain responses, in the up-regulation condition the cognitive change acted towards increasing pain processing, whereas cognitive load acted towards decreasing it. However, the relative impact of unspecific factors seemed to be more prominent than the influence of cognitive change. It should be noted here that not all clusters displayed the NOREG > UPREG > DWREG pattern of activations. Hence, more research on this topic is needed to further support this claim.

Although we ascribe the modulatory impact of our procedure to an increase in cognitive load, we failed to observe the increase in prefrontal activity that was hypothesized to be the source of modulatory effects. There is no certainty about the possible cause(s) that could account for the lack of this effect; however, this lack could pertain to some inherent limitations of the method used. As highlighted by one of the most comprehensive meta-analyses of pain neuroimaging studies, none of the analyzed EEG or MEG studies managed to record the activity of the PFC despite the consistent activation of this structure that was observed in fMRI studies (Apkarian et al., 2005).

The last issue that remains to be addressed concerns the question of why we did not observe a similar pattern of neural modulation to the one that was reported in studies that used an analogous reappraisal procedure that involved asking participants to actively up- or down-regulate their pain sensations by reinterpreting them (Fardo et al., 2015; Lapate et al., 2012; Woo et al., 2015). One possible explanation appeals to the method that was used to induce pain. While in our study pain was induced by phasic electric stimulation lasting 5 ms with eight stimuli in each session, in the study by Lapate et al. (2012) and Woo et al. (2015), the authors used painful heat to stimulate participants for approximately 12–12.5 seconds in each session. Putatively, this ultrashort and frequently repeated electric stimulation might have resulted in a distinct sense of pain that was less prone to reappraisal by means of reinterpretation. Conversely, Fardo et al. (2015) also used electric stimulation and observed up- and down-regulation of the neural response to pain according to the mental imagery instruction. Because this discrepancy is more difficult to explain, we suggest that future studies could include several different pain-induction techniques to shed light on this issue.

### Reappraisal, placebo, or hypnotic suggestion?

Our study was devoted to investigating the temporal dynamics of pain reappraisal, and our procedure was modeled after research on a situation-based cognitive reappraisal strategy (Ochsner et al., 2004, 2012). However, it is worth noting that our reappraisal task is somewhat reminiscent of other paradigms developed to study the impact of beliefs on the pain experience, such as anticipation/placebo or hypnotic suggestion. All of these forms aim to change cognitively the experience of one’s pain; however, in the case of placebo, this is typically achieved by more or less explicitly inducing expectations that pain is less (or more) negative than it really is (Ploghaus, 1999). Alternatively, in the case of hypnotic suggestion, it is achieved by encouraging hypnosis-prone participants to experience comfort and well-being while undergoing painful stimulation, utilizing as many as eight distinct suggestion-induction techniques, such as muscle relaxation and reality detachment (for more details on the hypnotic analgesia paradigm see Kiernan et al., 1995). Thus, both these forms rely heavily on environmental cues or externally generated expectations about pain. In contrast, the reappraisal strategy requires a more active approach towards one’s experience of pain instead of remaining a passive receiver of various (often quite elaborate, as in the case of hypnotic analgesia) forms of ready-made suggestions. This is one of reappraisal’s greatest assets, because it enables one to take control over the ongoing pain. Conversely, active engagement also is one of its greatest shortcomings, because it places the burden of influencing the pain experience on a suffering individual who may feel too psychologically overwhelmed to undertake any (cognitive) actions and/or may lack sufficient faith in their ability to influence pain sensations, which is required for reappraisal to be successful (Ford & Gross, 2018).

Despite the disparities, the resemblance between reappraisal and other pain-belief induction procedures, such as placebo or hypnotic suggestions, also evinces itself at the neural level (Ploghaus, Becerra, Borras, & Borsook, 2003; Tracey, 2010; van der Meulen et al., 2017). Notably, a recent fMRI study that compared placebo and cognitive reappraisal effects on processing of unpleasant visual material found that both procedures have a common anxiety-relieving effect that is correlated with attenuation of the amygdala and insula activity and engages partly overlapping subgenual cingulate-amygdala pathways to regulate emotions (Zhang, Guo, Zhang, & Luo, 2013). Another study extended these results to the pain domain, reporting a positive correlation between placebo-induced activity in the left dorsolateral PFC and a reduction in participants’ pain unpleasantness ratings and cognitive reappraisal ability scores (van der Meulen et al., 2017). Authors thus concluded that cognitive restructuring, which underlies reappraisal regulatory effects, may be a more common mechanism that mediates the effects of pain inhibition in placebo analgesia (van der Meulen et al., 2017). Although there are no direct comparisons between cognitive reappraisal and hypnotic suggestion, a recent meta-analysis provides a broadly similar picture of hypnosis and reappraisal neural effects, thus highlighting the role of the ACC as well as the prefrontal, insular, and somatosensory cortices (Del Casale et al., 2015). Here, however, the mechanisms that seem to account for pain reduction in hypnotic analgesia are somewhat different. Studies have shown that hypnotic analgesia is not only opiate-independent (which distinguishes it from the opiate-driven placebo effect), but also depends heavily on the degree of induced perception distortion/elevation of the perceptual threshold and the degree of hypnotic suggestibility (Del Casale et al., 2015). Conceivably, reinterpretation of painful somatosensory sensations plays a part in hypnoanalgesia as well, but this possibility is yet to be tested.

### Reliability of subjective ratings

A question remains: why did our procedure not affect the subjective ratings congruently with the observed activations of clusters, as suggested by neural signatures of pain processing? A convincing explanation would be the explicitness of the regulatory goal of our task. Although up-regulating pain sensations via reappraisal failed to enhance neural response in all but one brain region, participants reported feeling increased pain intensity and unpleasantness according to the provided instruction. We suggest that this apparent discrepancy between the neuronal and subjective measures might be a result of demand characteristics, which are not uncommon in reappraisal studies (Lieberman, Inagaki, Tabibnia, & Crockett, 2011). By having explicit knowledge about the goal of the regulatory task, participants can bias their ratings in a direction that is implicitly expected by the researchers. Furthermore, despite the neuronal or physiological evidence, some studies have shown that participants are less likely to report modulation of a subjective experience when the regulatory goal is hidden (Wang & Li, 2017; Wyczesany & Ligeza, 2017; Yuan, Ding, Liu, & Yang, 2015). It seems that tracking the subtle changes of an emotional state that has been evoked by moderately arousing and repeatedly presented affective stimuli poses a challenge to untrained or less emotionally reactive participants, who often bias their ratings based on their beliefs about what is expected of them.

### Limitations and directions for future research

Some limitations of our study should be mentioned. We examined only healthy female participants, because men and women respond differently to affective stimulation. We chose women, because they were shown to be more emotionally reactive than men but less prone to regulating automatically their emotions in response to affective stimulation (Domes et al., 2010; McRae, Ochsner, Mauss, Gabrieli, & Gross, 2008). Therefore, future studies could try to generalize our results in a sample of men. Moreover, it would be of an interest to examine the effects of reappraisal in a clinical population, especially chronic pain patients, as some initial results indicate that they could benefit from this kind of psychological intervention (Lawrence, Hoeft, Sheau, & Mackey, 2011). It also should be remembered that source localization using EEG methods inherently displays some limitations in terms of accuracy, so the conclusions regarding particular brain structures should be treated with some caution.

## Conclusions

This study investigated the temporal dynamics of the reappraisal effect on pain; we found robust evidence that reinterpreting nociceptive stimulation modulated the affective-evaluative aspect of pain processing in four functionally coupled limbic brain areas (ACC (90–145 ms), amygdala/ATmp (191–266 ms), OFC (188–288 ms)), which can be considered an early latency effect, and the insula (762–801 ms), which can be considered a late latency effect. This pattern of neural activity modulation indicates that reappraisal modulated pain processing at both the early perceptual (<300 ms) and the late cognitive-elaboration (>300 ms) stages, and the effects were evident as early as ~100 ms post stimulus onset. However, contrary to our expectations and to studies that used an analogous reappraisal procedure, i.e., asking participants to actively up- or down-regulate their pain sensations (Fardo et al., 2015; Lapate et al., 2012; Woo et al., 2015), we observed a general attenuation of neural response in one or both regulation conditions compared with the no-regulation condition, with no significant differences between them (except from the insula, for which activity was higher under up- vs. down-regulation).

Given the consistency of the neural modulation pattern, we argue that regulatory effects could be driven not only by a reappraisal-specific process of cognitive change, but also by other nonspecific factors that are known to reduce emotional responding. As reappraisal is one of the most cognitively complex forms of emotion regulation, one of the factors that significantly diminished emotional responding could have been the cognitive demand that accompanied the execution of an effortful reappraisal task. Hence, we found little evidence of effective up-regulation of pain experience at the neural level.

Subjective ratings, on the other hand, indicated successful up- and down-regulation according to the given instruction. However, given the fact that subjective ratings are strongly affected by demand characteristics and that demand characteristics are inherently associated with all reappraisal studies in which the study objective is not hidden from participants, we suggest that subjective results should be treated with caution.

To summarize, we found extensive evidence that reappraisal modulates pain processing from its earliest stages (~100 ms), but this modulation most probably reflects the joint effects of cognitive change and the unspecific cognitive load that accompanies the execution of the experimental task. For this reason, our study points to the importance of the inclusion of cautiously selected control conditions that allow incidental modulation of emotional responses by unspecific cognitive activity to be distinguished from the specific influence of cognitive change. It also raises the issue of demand characteristics that is common to all reappraisal studies in which the study objective is not hidden from participants.

### Open practices statement

**Statement 1.** The data on which the paper is based will be made available to reviewers upon mail request, but it cannot be made available to the general public as we did not collect participants’ permission for such practices.

**Statement 2.** This study was not preregistered.
